# Screening for single nucleotide variants, small indels and exon deletions with a next-generation sequencing based gene panel approach for Usher syndrome

**DOI:** 10.1002/mgg3.92

**Published:** 2014-06-15

**Authors:** Peter M Krawitz, Daniela Schiska, Ulrike Krüger, Sandra Appelt, Verena Heinrich, Dmitri Parkhomchuk, Bernd Timmermann, Jose M Millan, Peter N Robinson, Stefan Mundlos, Jochen Hecht, Manfred Gross

**Affiliations:** 1 Institute for Medical Genetics and Human Genetics, Charité Universitätsmedizin BerlinBerlin, Germany; 2 Department of Audiology and Phoniatrics, Charité Universitätsmedizin BerlinBerlin, Germany; 3 Max Planck Institute for Molecular GeneticsBerlin, Germany; 4 Unidad de Genetica, Hospital Universitario La Fe and CIBERERValencia, Spain; 5 Berlin Brandenburg Center for Regenerative Therapies BCRTBerlin, Germany

**Keywords:** Gene panel diagnostics, next-generation sequencing, Usher syndrome

## Abstract

Usher syndrome is an autosomal recessive disorder characterized both by deafness and blindness. For the three clinical subtypes of Usher syndrome causal mutations in altogether 12 genes and a modifier gene have been identified. Due to the genetic heterogeneity of Usher syndrome, the molecular analysis is predestined for a comprehensive and parallelized analysis of all known genes by next-generation sequencing (NGS) approaches. We describe here the targeted enrichment and deep sequencing for exons of Usher genes and compare the costs and workload of this approach compared to Sanger sequencing. We also present a bioinformatics analysis pipeline that allows us to detect single-nucleotide variants, short insertions and deletions, as well as copy number variations of one or more exons on the same sequence data. Additionally, we present a flexible in silico gene panel for the analysis of sequence variants, in which newly identified genes can easily be included. We applied this approach to a cohort of 44 Usher patients and detected biallelic pathogenic mutations in 35 individuals and monoallelic mutations in eight individuals of our cohort. Thirty-nine of the sequence variants, including two heterozygous deletions comprising several exons of USH2A, have not been reported so far. Our NGS-based approach allowed us to assess single-nucleotide variants, small indels, and whole exon deletions in a single test. The described diagnostic approach is fast and cost-effective with a high molecular diagnostic yield.

## Introduction

To date, 12 causative genes and a modifier gene have been identified for the three clinical subtypes of Usher syndrome (USH1, USH2, and USH3), whose clinical definition is based on the progression and severity of hearing impairment, vestibular dysfunction, and retinitis pigmentosa (RP) (Ebermann et al. 2010; Fiskerstrand et al. 2010; Richardson et al. 2011; Bonnet and El-Amraoui 2012; Puffenberger et al. 2012; Riazuddin et al. 2012). Although a clinical diagnosis of the Usher subtype is more difficult in early childhood due to the different age of onset of the symptoms, an early diagnosis is critical for appropriate educational and patient management choices.

At the time of our study, design works by Bonnet et al. (2011) and Le Quesne Stabej et al. (2012) had shown that ABI Sanger sequencing of all exons of nine genes that were known to be involved in the pathogenesis of Usher syndrome at that date had a diagnostic yield of about 80%. This required 578 primer reactions (Le Quesne Stabej et al. 2012) and sequencing of 366 exons (Bonnet et al. 2011), respectively, and the advent of new enrichment and next-generation sequencing (NGS) techniques offered the prospect of reducing labor and saving costs.

Up-to-date several studies have shown the feasibility of different enrichment and NGS approaches for the molecular diagnostics of Usher syndrome and demonstrated a comparable diagnostic yield as the above described ABI Sanger sequencing-based studies. To complement these works we describe an approach that is based on a customized SureSelect enrichment library from Agilent and Illumina sequencing and analyze the cost-benefits as well as the updateability of this approach.

In addition, we describe how the deep-sequencing data can be used to detect heterozygous exon deletions. This is of particular importance in Usher syndrome as a considerate number of cases with exon duplications and deletions have been reported for this disorder (Aller et al. 2010). Hitherto such intragenic rearrangements were detected with multiplex ligation-dependent probe amplification (MLPA) (Schouten et al. 2002) and the diagnostic procedure comprised MLPA as a second step if ABI Sanger sequencing was negative. In contrast to this sequential diagnostic procedure the NGS data allow the detection of single-nucleotide variants, small indels, and exonic deletions on a single dataset.

## Materials and Methods

In total, 42 unrelated European patients and two affected sisters (12-0880 and 12-0878 in Table 1) with Usher syndrome were included in this study. The clinical diagnosis was based on simultaneous occurrence of bilateral sensorineural hearing loss (HL) (HP:0000407) (Robinson et al. 2008) and bilateral RP (HP:0007703) (Robinson et al. 2008). The Charité University Medicine ethics board approved this study and informed consent for genetic testing was obtained from all probands and DNA was extracted from 200 μL of EDTA blood with a Biorobot M48 (Qiagen, Venlo, Netherlands) according to the manufacturer's protocol.

**Table 1:** Genotypes of 43 individuals with Usher syndrome

Patient	Gene	Effect on transcript	Effect on protein	Reference	Severity of hearing loss^1^	Age at diagnosis of hearing impairment (years)	Age at first symptoms of RP (years)	Vestibular dysfunction^2^
12-0652	USH2A	c.[11105G>A];[11105G>A]	p.(Trp3702*);(Trp3702*)	Glockle et al. (2014)	Severe	1	10	No
12-0653	USH2A	c.[1000C>T];[6805 + 2T>C]	p.(Arg334Trp)	Adato et al. (2000)	Profound	3	44	Unknown
12-0654	USH2A	c.[4628_4987del];[11864G>A]	p.(Trp3955*);(Gly1543_Pro1662del)	van Wijk et al. (2004), new	Moderate	7	21	No
12-0655	USH2A	c.[1558T>C]; [1558T>C]	p.(Cys520Arg);(Cys520Arg)	New	Unknown	Unknown	Unknown	Unknown
12-0656	USH2A	c.[3746C>T];[11864G>A]	p.(Pro1249Leu);(Trp3955*)	New, van Wijk et al. (2004)	Moderate	5	5	No
12-0657	USH2A	c.[7238A>G];[14131C>T]	p.(Asn2413Ser);(Gln4711*)	New, McGee et al. (2010)	Severe	1	13	No
12-0658	USH2A	c.[2610C>A];[8755A>C]	p.(Cys870*);(Thr2919Pro)	Le Quesne Stabej et al. (2012), new	Unknown	Unknown	Unknown	Unknown
12-0659	USH2A	c.2299delG	p.Glu767Serfs*21	Aller et al. (2006)	Severe	0	8	No
12-0660	USH2A	c.[2299delG];[11864G>A]	p.(Glu767Serfs*21);(Trp3955*)	Le Quesne Stabej et al. (2012), Aller et al. (2006)	Moderate	4	15	Yes
12-0661	USH2A	c.14074G>A	p.Gly4692Arg	Leijendeckers et al. (2009)	Moderate	50	40	No
12-0662	USH2A	c.2299delG	p.Glu767Serfs*21	Aller et al. (2006)	Moderate	2	Unknown	Unknown
12-0869	USH2A	[c.14131C>T];[14131C>T]	p.(Gln4711*);p.(Gln4711*)	Sandberg et al. (2008), new	Moderate	5	7	No
12-0870	USH2A	[c.11864G>A];[6806_7451del]	p.(Trp3955*);p.(Val2270Tyrfs*9)	van Wijk et al. (2004), new	Moderate	3	6	No
12-0871	USH2A	[c.2797C>T];[12703C>T]	p.(Gln933*);p.(Trp4235*)	Dreyer et al. (2008), new	Moderate	0	13	No
12-0872	CDH23	c.5131G>A	p.(Val1711Ile)	New	Mild	18	33	No
12-0873	MYO7A	c.[4400C>T];[6041A>G]	p.(Pro1467Leu);p.(His2014Arg)	New, new	Profound	0	39	Unknown
12-0875	USH2A	c.6084T>A	p.(Tyr2028*)	New	Moderate	4	16	No
12-0876	PCDH15	c.400C>G	p.Arg134Gly	New	Profound	1	30	No
12-0877	USH2A	[c.923_924insGCCA];[1673_1677del]	p.(His308Glnfs*16);p.(Asp558Alafs*6)	Dreyer et al. (2008), new	Severe	1	16	No
12-0878	USH2A	[c.10939G>A];[7139_7140del]	p.(Gly3647Ser);p.(Leu2380Profs*37)	New, new	Severe	6	9	No
12-0879	USH2A	[c.11864G>A];[1036A>C]	p.(Trp3955*);p.(Asn346His)	van Wijk et al. (2004), Dreyer et al. (2008)	Moderate	4	18	No
12-0880	USH2A	[c.10939G>A];[7139_7140del]	p.(Gly3647Ser);p.(Leu2380Profs*37)	New, new	Moderate	3	12	No
12-0881	GPR98	c.3974C>T	p.(Thr1325Met)	New	Moderate	9	8	No
12-0882	USH2A	[c.2299delG];[11831C>A]	p.(Glu767Serfs*21);(Ala3944Asp)	Aller et al. (2006), new	Moderate	3	11	No
12-0884	USH2A	[c.11864G>A];[7524delT]	p.(Trp3955*);(Phe2508 fs)	van Wijk et al. (2004), new	Moderate	10	8	No
12-0885	USH2A	[c.11864G>A];[1036A>C]	p.(Trp3955*);p.(Asn346His)	Dreyer et al. (2008), van Wijk et al. (2004)	Moderate	2	10	No
12-0886	USH2A	[c.11713C>T];[1808_1810del]	p.(Arg3905Cys);p.(Gly603del)	New	Moderate	9	Unknown	No
12-0887	USH2A	[c.9424G>T];[2299delG]	p.(Gly3142*); p.(Glu767Serfs*21)	Aller et al. (2006), Baux et al. (2008)	Severe	7	6	No
12-0888	USH2A	[c.2299delG];[2299delG]	p.(Glu767Serfs*21)	Aller et al. (2006)	Severe	4	6	No
12-0889	USH1G	[c.143T>C];[186_187del]	p.(Leu48Pro);p.(Ile63Leufs*71)	New	Profound	0	15	Yes
12-0890	USH2A	[c.100C>T];[12790G>T]	p.(Arg34*);p.(Glu4264*)	Dreyer et al. (2008), New	Profound	4	18	Unknown
12-0891	USH2A	[c.5153A>C];[7595-2144A>G]	p.(Gln1718Pro)	Vache et al. (2012)	Moderate	1	16	No
12-0892	USH2A	[c.920_923dup];[11864G>A]	p.(His308Glnfs*16);p.(Trp3955*)	Kamphans and Krawitz (2012), Pollard et al. (2010)	Moderate	2	25	No
12-0893	USH1C	c.2611G>A	p.(Ala871Thr)	New	Moderate	40	28	No
12-0894	USH2A	[c.12284G>A];[2610C>A]	p.(Gly4095Asp);p.(Cys870*)	New, Le Quesne Stabej et al. (2012)	Severe	4	19	No
12-0895	MYO7A	[c.93C>A];[3862G>C]	p.(Cys31*); p.(Ala1288Pro)	Weston et al. (1996), Janecke et al. (1999)	Profound	0	9	No
12-0903	USH2A	[c.4756C>T];[5607_5615del]	p.(Gln1586*);p.(Arg1870_Ala1872del)	New	Moderate	0	33	Unknown
12-0906	MYO7A	[c.2005C>T];[3862G>C]	p.(Arg669*);p.(Ala1288Pro)	Janecke et al. (1999)	Profound	0	13	Yes
12-0909	USH2A	[c.9871G>A];[5975A>G]	p.(Gly3291Ser);p.(Tyr1992Cys)	New, McGee et al. (2010)	Profound	0	13	No
12-0910	GRP98	[c.17488C>T];[18310G>T]	p.(Gln5830*);p.(Glu6104*)	New	Moderate	3	20	No
13-0975	USH2A	[c.8063C>T];[7342C>G]	p.(Ser2688Phe);p.(Pro2448Ala)	New	Severe	5	5	No
13-0976	USH2A	[c.14131C>T];[11864G>A]	p.(Gln4711*);p.(Trp3955*)	van Wijk et al. (2004), Sandberg et al. (2008)	Severe	33	30	No
13-1008	USH2A	[c.923_924insGCCA];[923_924insGCCA]	p.(His308Glnfs*16);p.(His308Glnfs*16)	New	Severe	4	29	No

Biallelic mutations were identified in 36 individuals in *USH2A* (transcript NM_206933.2), *MYO7A* (transcript NM_000260.3), *GRP98* (transcript NM_032119.3). Additionally, monoallelic mutations, also in *PCDH15* (transcriptNM_033056.3), *USH1C* (transcriptNM_153676.3), and *CDH23* (transcriptNM_022124.2), were identified in eight individuals. All the listed novel mutations were predicted as pathogenic or likely pathogenic.

New, novel variant, first described in this article.

1 Severity of hearing loss based on the convent of EU Concerted Action on Genetics of Hearing Impairments (H.E.A.R.), applied to the better hearing ear, averaged across 500, 1000, 2000, and 4000 Hz (Pure tone audiometry, PTA): mild 20–40 dB HL, moderate 41–70 dB HL, severe 71–95 dB HL, profound >95 dB HL.

2 Vestibular dysfunction evaluated by caloric test and/or anamnestic delayed motor milestones (walking alone >20 months).

We designed a customized SureSelect oligonucleotide library (Agilent, Santa Clara, CA) for the targeted enrichment of all known exons of the Usher genes *CDH23*, *CLRN1*, *DFNB31*, *GPR98*, *MYO7A*, *PCDH15*, *USH1C*, *USH1G*, *USH2A*, and additional genes that are associated with nonsyndromic hearing impairment (https://gene-talk.de/gene_sets/219).

The recently identified causal Usher genes *ABHD12* (Fiskerstrand et al. 2010), *CIB2* (Riazuddin et al. 2012), and *HARS* (Puffenberger et al. 2012) as well as the gene *PDZD7* (Ebermann et al. 2010) that is discussed as a modifier in Usher syndrome, could not be included at the time of the bait design and were not part of the NGS analysis. Genomic DNA of all patients was enriched for this target region according to the manufacturer's protocol, followed by single-read cluster generation on a Cluster Station (Illumina, San Diego, CA). The captured, purified, and clonally amplified library was then sequenced on a HiSeq 2500 (Illumina) and mapped to the human reference sequence GRCh37, resulting in a mean coverage of above 300-fold for all exons and more than a 10-fold coverage for more than 95% of the target region. Variants were detected with SAMtools (Li 2011) after removing duplicate reads and annotated with ANNOVAR (Wang et al. 2010). Variants were further filtered in GeneTalk (Kamphans and Krawitz 2012) with the public Usher syndrome gene panel (https://gene-talk.de/gene_sets/437). Alleles that occur with a frequency above 1% in the 1000 genomes project (Pollard et al. 2010) and that did not have an effect on the protein level were removed. Mutations that were not yet listed in the human genome mutation database or the locus specific database (LSDB) were subjected to MutationTaster (Schwarz et al. 2010). Novel missense variants, classified as deleterious were also assessed using Usher Syndrome Missense Analysis (https://neuro-2.iurc.montp.inserm.fr/USMA), a web-based tool dedicated to analysis of missense variants in Usher genes (Besnard et al. 2013).

**Figure 1:**
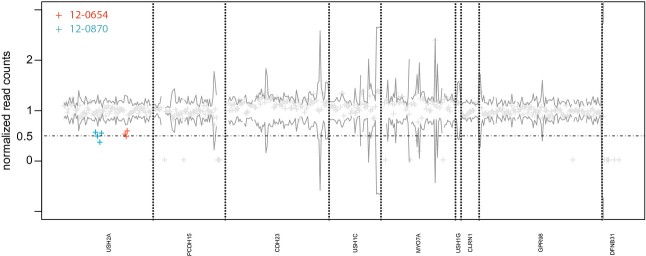
Detection of exon deletions by sequence coverage. The exon coverage was corrected by the total read count per sample. The variance of the sequence coverage depends on the exon. The black line indicates 2 SD for the normalized coverage per exon that is based on all samples. In sample 12-0654 the three consecutive exons 22–24 of *USH2A*, and in sample 12-0870 the four consecutive exons 38-41 of *USH2A* had a normalized read count around 0.5 indicating heterozygous deletions. The two heterozygous deletions NM_206933.2:c.4628_4987del and c.6806_7451del could be confirmed by qPCR and multiplex ligation-dependent probe amplification.

For the detection of exon deletions, we first counted the reads per exon and normalized this value for each sample by the total number of reads that were mapped to the target region. This normalized read count per exon was used to compute the mean and variance for the coverage per exon in all analyzed samples. Exons with a normalized coverage around 0.5 and below 2 SD of the mean were classified as heterozygously deleted and further analyzed by qPCR and MLPA. Java and R code that can be used to derive the read counts from bam files as well as the normalized exon-specific coverage are provided as supporting information (bam2readcounts.zip). The short-read alignments that are required to reproduce Figure 1 are provided for download as bam files at: https://compbio.charite.de/contao/tl_files/groupmembers/pkrawitz/bam/.

## Results

Forty-two unrelated individuals and two siblings (12-0878, 12-0880) with Usher syndrome were screened for mutations in the Usher genes *CDH23*, *CLRN1*, *DFNB31*, *GPR98*, *MYO7A*, *PCDH15*, *USH1C*, *USH1G*, and *USH2A*. Biallelic mutations could be identified in 35 individuals and monoallelic mutations in eight individuals. In total, we identified 52 distinct, pathogenic, or likely pathogenic mutations in our cohort, 23 missense, 15 nonsense, 2 splicing mutations, and 10 small insertions or deletions (Table 1). In two cases we could identify heterozygous deletions that comprised exons 24–26, c.4628_4987del (patient 12-0654 in Table 1) and exons 38-41, c.6806_7451del (patient 12-0870 in Table 1). The deletions that we detected due to reduced sequence coverage could be validated by qPCR and MLPA (supporting information).

Thirty-six of these sequence variants have not been previously described in the literature and were not presented in the LSDB for USH genes (Roux et al. 2011) (Table 1, Fig. 1). Among these new findings were mutations in seven different Usher genes, *CDH23*, *GPR98*, *MYO7A*, *PCDH15*, *USH1G*, *USH1C*, and *USH2A*. All novel variants were assessed based on a classification system for variants of uncertain clinical significance described by David Baux et al. (2013) that relies on the practice guidelines for the interpretation and reporting of unclassified variants in clinical molecular genetics from the Clinical and Medical Genetics Society. For missense mutations altogether six criteria from biological observations and in silico predictions are considered to compute a score that guides the classification. The biological criteria comprise presence in the literature and databases, frequency in control DNAs, and allelic position. Especially in recessive diseases such as Usher syndrome the situation of the other allele may be informative for the classification. A proven deleterious variant in trans to a novel variant, for example, affects the argument in favor for pathogenicity.

The in silico approaches use orthologous sequence alignments and domain alignments for the assessment of a mutated position as well as predictions about the structural effects of an amino acid exchange. Finally the total score was used to classify the variant of unknown significance as certainly benign, likely benign, likely pathogenic, or certainly pathogenic and all our novel variants that are listed in Table 1 were classified as likely or certainly pathogenic.

The severity of the hearing impairment, the age of onset of HL and RP, and the status of the vestibular function (VD) are also listed in Table 1 for each patient. A classification into one of the three Usher subtypes could be based on these clinical data (USH1: profound HL and RP before puberty and often VD, USH2: moderate to severe HL and RP with onset after puberty, USH3: progressive HL with variable RP). However, the classification into three subtypes that was thought to correspond to mutations in specific genes is gradually eroding in recent years. On one hand the phenotypic variability in identical mutations even in similar genetic backgrounds is surprisingly high as the example of two affected sisters in our cohort illustrates: 12-0878 and 12-0880 are both compound heterozygous for a newly identified missense mutation, p.(Gly3647Ser), and a frame shifting 1 bp deletion, p.(Leu2380Profs*37). Although having the same pathogenic alleles the hearing impairment in one of the sisters is more severe and the onset of HL and RP differ by 3 years.

On the other hand it is yet not clear to which degree gene dosage, di- or oligo-genic effects of pathogenic alleles contribute to the severity of the clinical symptoms. While we did observe novel variants, for example, in *USH2A* in patients that fit to USH2 (e.g., 12-0894, 12-0654) and novel variants, for example, in *USH1G* that fit to USH1 (12-0889), other cases cannot be properly classified into these subtypes. Especially the monoallelic variants that we identified in *CDH23* and *USH1C* which are genes formerly associated with subtype USH1, show only mild to moderate HL (12-0872, 12-0893). At the current moment it is not clear whether the monoallelic variants can explain the milder phenotypes alone or whether the second hits were simply missed due to technological limitations. Six patients in whom we detected only one heterozygous, monoallelic mutation in *USH2A* were subjected to Sanger sequencing for the deep intronic, pathogenic mutation c.7595-2144A>G and a second heterozygous hit was detected in one of these samples (12-0891) (Vache et al. 2012). Meta-analyses of larger cohorts are needed for a statistically sound analysis of further genotype–phenotype correlations.

## Discussion

The molecular diagnosis of Usher syndrome is challenging due to the genetic heterogeneity and the large size and variability in some of the Usher genes. Although mutations in 12 genes have been described to cause Usher syndrome, the most common subtype of Usher syndrome remains USH2 and mutations in *USH2A* have been identified in the majority of the reported cases (van Wijk et al. 2004; Dreyer et al. 2008). The detection of *USH2A* mutations in 35 of our 44 analyzed individuals is therefore in accordance with these findings.

To date, two large studies have analyzed all coding exons of Usher genes by Sanger sequencing (Bonnet et al. 2011; Le Quesne Stabej et al. 2012). These studies showed that the comprehensive analysis of all Usher genes improves the diagnostic yield. Especially the high proportion of family-specific pathogenic mutations showed that the use of microarray-based approaches in the molecular diagnosis of Usher syndrome is limited. In our study, almost half of the mutations have not been previously described in the literature, confirming a high rate of novel variants in Usher syndrome.

Steele-Stallard et al. (2013) screened Usher cases in which only monoallelic mutations were identified by Sanger sequencing of *USH2A* for duplications and known pathogenic deep intronic mutations. By this additional diagnostic approach they could identify the second pathogenic mutation in about a third of the cases. If we consider only single-nucleotide variants and small indels in *USH2A* in our cohort, we had seven monoallelic cases (12-0654, 12-0659, 12-0661, 12-0662, 12-0870, 12-0875, and 12-0891). However, in two of these cases the second mutations were deletions of several exons that we identified based on their reduced sequence coverage (12-0654 and 12-0870). In one case we also identified the deep intronic mutation c.7593-2144A>G (12-0891). Our results confirm the findings of Steele-Stallard that the rate of biallelic detection can be increased in Usher patients with an analysis pipeline that does not only consider coding variants and variants that affect the canonic splice site. It might be speculated that additional deeply intronic variants that affect splicing as well as new Usher genes will even further increase the rate of biallelic pathogenic findings in Usher patients. Baits for these new target regions may then be included into the described enrichment protocol.

In summary, we describe in this work a comprehensive NGS-based approach and bioinformatics data processing with a gene panel that is updateable in silico for the molecular diagnostics of Usher syndrome. The feasibility of target enrichment and massively parallel sequencing for comprehensive testing of gene panels was already described for nonsyndromic hereditary HL, as well as Usher syndrome by other groups (Shearer et al. 2010; Licastro et al. 2012; Besnard et al. 2013).

Shearer et al. (2010) were the first to describe the targeted enrichment and sequencing by SureSelect-Illumina and NimbleGen-Roche-454 methods for 1258 genomic regions of 54 genes comprising also the nine Usher genes *MYO7A*, *USH1C*, *CDH23*, *PCDH15*, *USH1G*, *USH2A*, *GPR98*, *DFNB31*, and *CLRN1* and assessed the sensitivity of these approaches in 10 test samples. Licastro et al. (2012) compared the effectiveness of exome sequencing compared to long PCR-based methods that both include the exons of the above named nine Usher genes and showed that the coverage of long PCR-based approach is currently still superior to standard human all exon kits. Besnard et al. (2013) evaluated in an extensive study, including 71 patients, the sensitivity of a NimbleGen-Roche Junior-based approach as a clinical test.

With a SureSelct/Illumina-based enrichment and sequencing approach, we achieved a similar diagnostic yield in Usher syndrome with deep short-read sequence data as compared to standard approaches that are based on Sanger sequencing followed by MLPA or NGS-based approaches. Compared to Sanger sequencing this simplifies the workflow, speeds up the diagnostic procedure, and might also help to save costs.

For the enrichment we used customized oligonucleotide baits in solution from Agilent (SureSelect). The great advantage of this approach compared to Sanger sequencing gene by gene is the reduction of hands on time in the workflow and the parallelization of the sequencing on the Illumina platform.

However, if new genes are identified in context of a heterogeneous disorder such as Usher syndrome, a revised version of enrichment baits has to be designed and ordered. That often means that recently identified genes have to be sequenced by Sanger, until they are included into the enrichment approach in a newer version of the bait library. For this reason the genes *HARS*, *ABHD12*, *CIB12* as well as the modifier gene *PDZD7* were not yet included in our current study and have to be added to a revised version of our bait library.

Only whole exome sequencing (WES) would allow us to reanalyze a dataset with a gene panel that is updated in silico, such as https://gene-talk.de/gene_sets/437. However, besides the lower performance of WES regarding the coverage of the Usher target region that was already discussed by Licastro et al., also the reduced costs of sequencing a targeted gene panel might outweigh the easy in silico updateability of the analysis of WES data in a diagnostic setting. While an exome with an average coverage of 60× costs between 800 and 1200 Euro, a targeted gene panel approach might be more affordable.

Kits for enrichment and sequencing make up the majority of the costs for consumables. The price for the enrichment reactions decreases considerably when large quantities are ordered and costs of 150–200 Euro per sample are reasonable if more than 100 reactions are bought.

The sequencing costs depend mainly on the choice of the sequencing platform. While, for example, a 100 bp paired-end run on the HiSeq and a 150 bp paired-end run on the MiSeq both cost about 1000 Euro, the raw sequence output on a HiSeq lane is about 5–10 times higher.

This means with a raw sequence output of 20 GB per lane, a planned coverage of 100× and a target region of less than 500 kb, there would be room for 200 samples assuming an enrichment factor of 0.5. The limiting factor in such a case is rather the ability to barcode and pool the samples equimolarly on a single lane than the mean raw sequencing output to reach a minimum coverage per sample. When a standard barcode set of 24 different indices is used, the sequencing costs on a HiSeq and MiSeq might be as low as 50 Euros per sample.

Besides the consumables the investment costs for an NGS platform can also be broken down to a single sample. If we assume 160 samples per HiSeq run and 20 such runs per year, or 140 such runs in a period of 7 years, which is a common assumption for the period of amortization, and if we assume 500,000 Euro for the purchase price of a HiSeq, then we have about 20 Euro per sample.

If we also consider labor this will make the major contribution to our cost account. The difficulty here is to estimate the scaling factor for each working step if the number of samples increases. The ultrasonic fragmentation of the DNA, for example, is a working step that scales almost linearly with the number of samples. Ten samples will require roughly 10 times longer, as the Covaris, the device that we are using, is basically limited to one tube. However, most procedures that require pipetting can be done more efficiently for several samples if processed in parallel, as, for example, multichannel pipettes may be used.

In the following, we therefore assume the time that is required by a technician to process a single sample. The library preparation takes about 6 h, the enrichment and capturing about 4.5 h, and the sequencing on a MiSeq about 1.5 h adding up to a total hands on time of 12 h. In these calculations the number of exons that are processed is irrelevant as a single in solution oligonucleotide bait library is used.

If we compare that work load to ABI Sanger sequencing of 500 exons, we would account around 32 h for the polymerase chain reactions and their clean up followed by 32 h for the sequencing reactions. If we assume an annual gross wage of 45,000 Euro for a lab technician and if we assume that about 1500 h are spend productively this translates to about 30 Euro per hour labor. With the estimated labor costs for a technician this corresponds to more than 30 × 50 = 1500 Euros that can be saved per sample by the NGS approach that we described.

Besides the efficiency and cost considerations we also analyzed the prerequisites for the sequencing data to detect exon deletions. We choose an approach to identify exon deletions by detecting significant reductions in sequence coverage. There are several parameters that crucially influence the expected coverage in a wild-type sample. Besides the total amount of raw sequence data that is (1) the enrichment efficiency that is exon specific and (2) the size distribution and sequencing strategy of the DNA fragments. The easiest and most effective way to deal with the exon-specific enrichment and sequencing efficiencies is to compare only the coverage of the same exon that was obtained by the same enrichment library in other samples. The choice of the sequencing protocol and the fragment length mainly influence the amount of sequencing data that map into the neighboring introns. In a paired-end run a high proportion of the short reads will be intronic if the sequenced fragment excels the exon size.

However, even if we considered only the coverage of the coding positions the variance for the normalized exon-specific coverage was markedly increased, if samples of different experiments were pooled. This indicates that the fragment size also influences the sequencing efficiency and the expected coverage of the exons. The variability in the size distribution of the DNA fragments in our protocol might be due to slightly varying times of ultrasound shearing. Although it might be interesting to analyze whether the expected coverage for exon amplicons from multiplex PCRs is more robust than with an enrichment approach, there is a much simpler way to deal with it. The variance of the normalized exon-specific coverage for diploid samples was low as long as we pooled only samples of the same enrichment, fragmentation, and sequencing experiment. Not surprisingly this is also the common approach for array-cgh experiments, where a control DNA is used in any experiment.

## Supporting Information

**Figure S1.** Short-sequence read alignments visualized with igv. Red arrows in upper panel indicate reduced coverage of USH2A exons in patient 1598. **Figure S2.** Patient 1598 is heterozygous at chr1:216.348.744C>T (rs1805049). This SNP is in exon 21 of USH2A (NM_206933). Exon 21 is thus not deleted in patient 1598. **Figure S3.** Patient 1598 is heterozygous at chr1:216258194G>T (rs56110889). This SNP is in exon 25 of USH2A (NM_206933). Thus, exon 25 not deleted in patient 1598. **Figure S4.** Heterozygous deletions of exons 22–24 in patient 1598 but not in control 1602. Exon 27 is not deleted in 1598 and 1602. **Table S1.** qPCR primers for USH2A.

## References

[b1] Adato A, Weston MD, Berry A, Kimberling WJ, Bonne-Tamir A (2000). Three novel mutations and twelve polymorphisms identified in the USH2A gene in Israeli USH2 families. Hum. Mutat.

[b2] Aller E, Jaijo T, Beneyto M, Najera C, Oltra S, Ayuso C (2006). Identification of 14 novel mutations in the long isoform of USH2A in Spanish patients with Usher syndrome type II. J. Med. Genet.

[b3] Aller E, Jaijo T, Garcia-Garcia G, Aparisi MJ, Blesa D, Diaz-Llopis M (2010). Identification of large rearrangements of the PCDH15 gene by combined MLPA and a CGH: large duplications are responsible for Usher syndrome. Invest. Ophthalmol. Vis. Sci.

[b4] Baux D, Faugere V, Larrieu L, Le Guedard-Mereuze S, Hamroun D, Beroud C (2008). UMD-USHbases: a comprehensive set of databases to record and analyse pathogenic mutations and unclassified variants in seven Usher syndrome causing genes. Hum. Mutat.

[b8] Baux D, Vache C, Malcolm S, Claustres M, Roux A-F (2013). Interpretation of variants of uncertain clinical significance (VUCS): the paradigm of Usher syndrome.

[b5] Besnard T, García-García G, Baux D, Vaché C, Faugère V, Larrieu L (2013). Experience of targeted Usher exome sequencing as a clinical test. Mol. Genet. Genomic Med.

[b6] Bonnet C, El-Amraoui A (2012). Usher syndrome (sensorineural deafness and retinitis pigmentosa): pathogenesis, molecular diagnosis and therapeutic approaches. Curr. Opin. Neurol.

[b7] Bonnet C, Grati M, Marlin S, Levilliers J, Hardelin JP, Parodi M (2011). Complete exon sequencing of all known Usher syndrome genes greatly improves molecular diagnosis. Orphanet J. Rare Dis.

[b9] Dreyer B, Brox V, Tranebjaerg L, Rosenberg T, Sadeghi AM, Moller C (2008). Spectrum of USH2A mutations in Scandinavian patients with Usher syndrome type II. Hum. Mutat.

[b10] Ebermann I, Phillips JB, Liebau MC, Koenekoop RK, Schermer B, Lopez I (2010). PDZD7 is a modifier of retinal disease and a contributor to digenic Usher syndrome. J. Clin. Investig.

[b11] Fiskerstrand T, H'Mida-Ben Brahim D, Johansson S, M'Zahem A, Haukanes BI, Drouot N (2010). Mutations in ABHD12 cause the neurodegenerative disease PHARC: an inborn error of endocannabinoid metabolism. Am. J. Hum. Genet.

[b12] Glockle N, Kohl S, Mohr J, Scheurenbrand T, Sprecher A, Weisschuh N (2014). Panel-based next generation sequencing as a reliable and efficient technique to detect mutations in unselected patients with retinal dystrophies. Eur. J. Hum. Gen.

[b13] Janecke AR, Meins M, Sadeghi M, Grundmann K, Apfelstedt-Sylla E, Zrenner E (1999). Twelve novel myosin VIIA mutations in 34 patients with Usher syndrome type I: confirmation of genetic heterogeneity. Hum. Mutat.

[b14] Kamphans T, Krawitz PM (2012). GeneTalk: an expert exchange platform for assessing rare sequence variants in personal genomes. Bioinformatics.

[b15] Le Quesne Stabej P, Saihan Z, Rangesh N, Steele-Stallard HB, Ambrose J, Coffey A (2012). Comprehensive sequence analysis of nine Usher syndrome genes in the UK National Collaborative Usher Study. J. Med. Genet.

[b16] Leijendeckers JM, Pennings RJ, Snik AF, Bosman AJ, Cremers CW (2009). Audiometric characteristics of USH2a patients. Audio. Neurootol.

[b17] Li H (2011). A statistical framework for SNP calling, mutation discovery, association mapping and population genetical parameter estimation from sequencing data. Bioinformatics.

[b18] Licastro D, Mutarelli M, Peluso I, Neveling K, Wieskamp N, Rispoli R (2012). Molecular diagnosis of Usher syndrome: application of two different next generation sequencing-based procedures. PLoS One.

[b19] McGee TL, Seyedahmadi BJ, Sweeney MO, Dryja TP, Berson EL (2010). Novel mutations in the long isoform of the USH2A gene in patients with Usher syndrome type II or non-syndromic retinitis pigmentosa. J. Med. Genet.

[b20] Pollard KS, Hubisz MJ, Rosenbloom KR, Siepel A (2010). Detection of nonneutral substitution rates on mammalian phylogenies. Genome Res.

[b21] Puffenberger EG, Jinks RN, Sougnez C, Cibulskis K, Willert RA, Achilly NP (2012). Genetic mapping and exome sequencing identify variants associated with five novel diseases. PLoS One.

[b22] Riazuddin S, Belyantseva IA, Giese AP, Lee K, Indzhykulian AA, Nandamuri SP (2012). Alterations of the CIB2 calcium- and integrin-binding protein cause Usher syndrome type 1J and nonsyndromic deafness DFNB48. Nat. Genet.

[b23] Richardson GP, de Monvel JB, Petit C (2011). How the genetics of deafness illuminates auditory physiology. Annu. Rev. Physiol.

[b24] Robinson PN, Kohler S, Bauer S, Seelow D, Horn D, Mundlos S (2008). The Human Phenotype Ontology: a tool for annotating and analyzing human hereditary disease. Am. J. Hum. Genet.

[b25] Roux AF, Faugere V, Vache C, Baux D, Besnard T, Leonard S (2011). Four-year follow-up of diagnostic service in USH1 patients. Invest. Ophthalmol. Vis. Sci.

[b26] Sandberg MA, Rosner B, Weigel-DiFranco C, McGee TL, Dryja TP, Berson EL (2008). Disease course in patients with autosomal recessive retinitis pigmentosa due to the USH2A gene. Invest. Ophthalmol. Vis. Sci.

[b27] Schouten JP, McElgunn CJ, Waaijer R, Zwijnenburg D, Diepvens F, Pals G (2002). Relative quantification of 40 nucleic acid sequences by multiplex ligation-dependent probe amplification. Nucleic Acids Res.

[b28] Schwarz JM, Rodelsperger C, Schuelke M, Seelow D (2010). MutationTaster evaluates disease-causing potential of sequence alterations. Nat. Methods.

[b29] Shearer AE, DeLuca AP, Hildebrand MS, Taylor KR, Gurrola J, Scherer S (2010). Comprehensive genetic testing for hereditary hearing loss using massively parallel sequencing. Proc. Natl. Acad. Sci. USA.

[b30] Steele-Stallard HB, Le Quesne Stabej P, Lenassi E, Luxon LM, Claustres M, Roux AF (2013). Screening for duplications, deletions and a common intronic mutation detects 35% of second mutations in patients with USH2A monoallelic mutations on Sanger sequencing. Orphanet J. Rare Dis.

[b31] Vache C, Besnard T, le Berre P, Garcia-Garcia G, Baux D, Larrieu L (2012). Usher syndrome type 2 caused by activation of an USH2A pseudoexon: implications for diagnosis and therapy. Hum. Mutat.

[b32] Wang K, Li M, Hakonarson H (2010). ANNOVAR: functional annotation of genetic variants from high-throughput sequencing data. Nucleic Acids Res.

[b33] Weston MD, Kelley PM, Overbeck LD, Wagenaar M, Orten DJ, Hasson T (1996). Myosin VIIA mutation screening in 189 Usher syndrome type 1 patients. Am. J. Hum. Genet.

[b34] van Wijk E, Pennings RJ, te Brinke H, Claassen A, Yntema HG, Hoefsloot LH (2004). Identification of 51 novel exons of the Usher syndrome type 2A (USH2A) gene that encode multiple conserved functional domains and that are mutated in patients with Usher syndrome type II. Am. J. Hum. Genet.

