# Supplementary material for: Screening for single nucleotide variants, small indels and exon deletions with a next-generation sequencing based gene panel approach for Usher syndrome
Source: Mol Genet Genomic Med. 2014 Jun 15;2(5):393–401. doi: 10.1002/mgg3.92 (PMC4190874; doi:10.1002/mgg3.92)
Supplement: Supplementary file 1 [file mgg30002-0393-SD1.pdf]

Supplementary Information

in the following samples 1598, 1602 represent samples 12-0654, 12-0658 of table 1 of the main manuscript

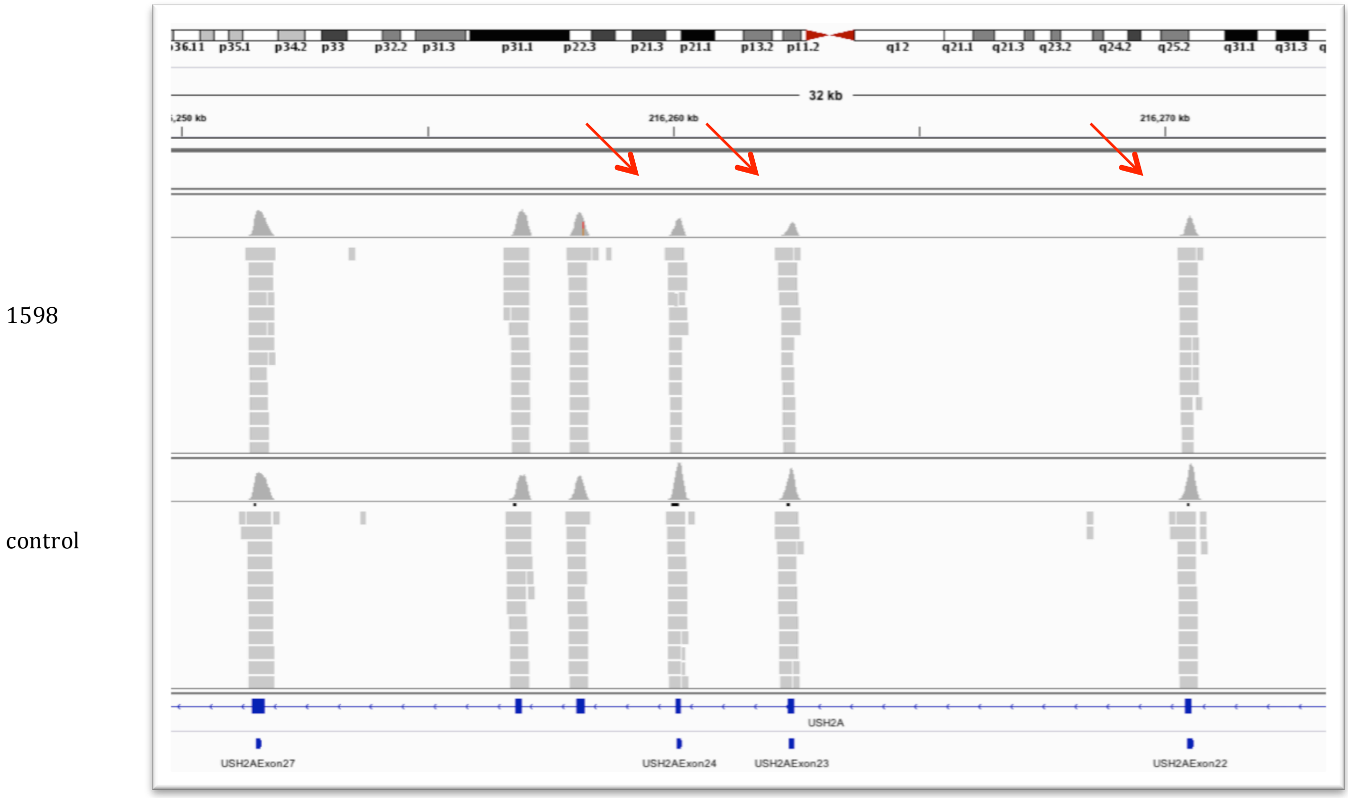

Supplementary Figure 1: short sequence read alignments visualized with igv. Red arrows in upper panel indicate reduced coverage of USH2A exons in patient 1598.

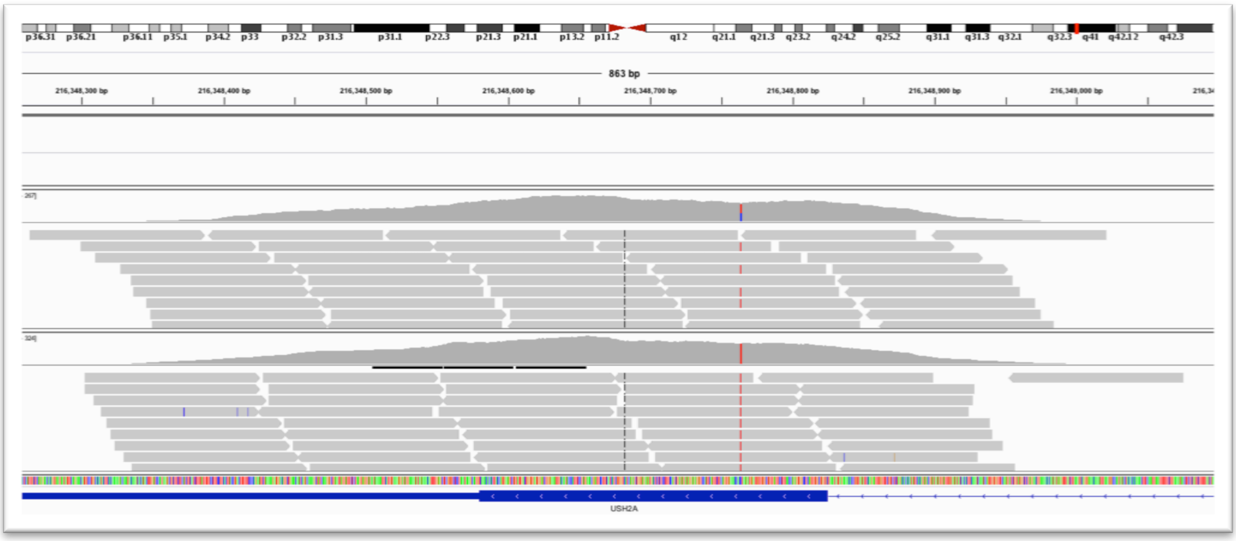

Supplementary Figure 2: patient 1598 is heterozygous at chr1:216.348.744C>T (rs1805049). This SNP is in Exon 21 of USH2A (NM_206933). Exon 21 is thus not deleted in patient 1598.

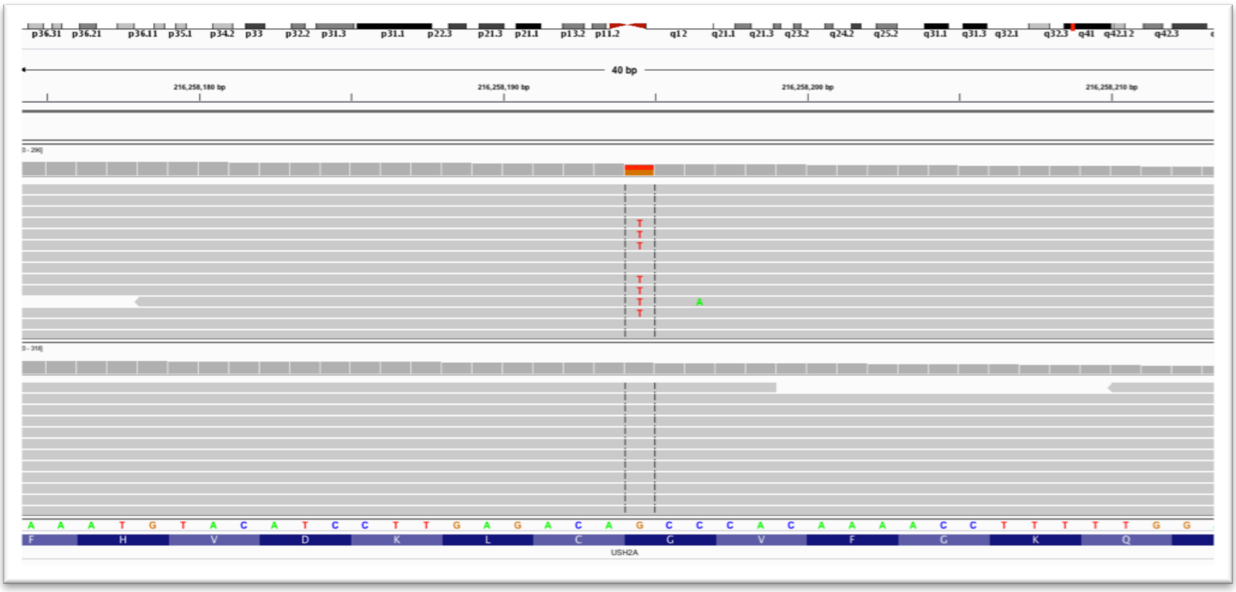

Supplementary Figure 3: Patient 1598 is heterozygous at chr1:216258194G>T (rs56110889). This SNP is in Exon 25 of USH2A (NM_206933). Thus Exon 25 not deleted in patient 1598.

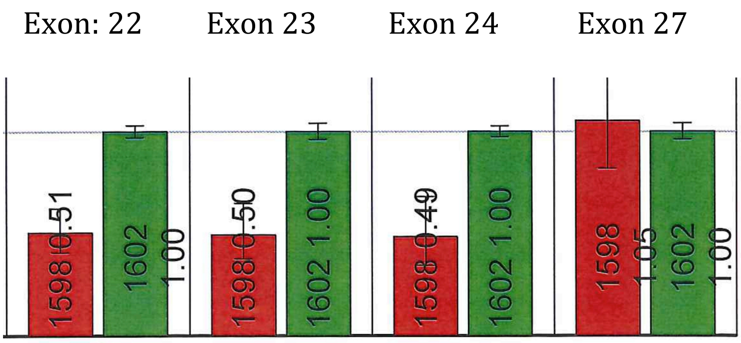

Supplementary Figure 4: Heterozygous deletions of exons 22-24 in patient 1598 but not in control 1602. Exon 27 is not deleted in 1598 and 1602.

**Exon 22 fw**	ccctcccagaaagactcctg
**Exon 22 rv**	tgtatttctcatctcctcttctctagg
**Exon 23 fw**	caaaagcctgatgcctaatagc
**Exon 23rv**	ggggtcaccagtggaagtaac
**Exon 24 fw**	ccaggtgatgctgcaaagac
**Exon 24 rv**	aaaaccccagcactgatttg
**Exon 25 fw**	cgatgcatgcttcatcagtc
**Exon 25 rv**	gctatcctattgtaatggaaagtgg

Supplementary Table 1: qPCR Primers for USH2A
